# Within-patient plasmid dynamics in *Klebsiella pneumoniae* during an outbreak of a carbapenemase-producing *Klebsiella pneumoniae*

**DOI:** 10.1371/journal.pone.0233313

**Published:** 2020-05-18

**Authors:** Joep J. J. M. Stohr, Jaco J. Verweij, Anton G. M. Buiting, John W. A. Rossen, Jan A. J. W. Kluytmans

**Affiliations:** 1 Laboratory for Medical Microbiology and Immunology, Elisabeth-TweeSteden Hospital, Tilburg, The Netherlands; 2 Department of Infection Control, Amphia Hospital, Breda, The Netherlands; 3 Department of Medical Microbiology and Infection Prevention, University of Groningen, University Medical Center Groningen, Groningen, The Netherlands; 4 Department of Pathology, University of Utah School of Medicine, Salt Lake City, UT, United States of America; 5 Julius Center for Health Sciences and Primary Care, University Medical Center Utrecht, Utrecht University, Utrecht, The Netherlands; 6 Amphia Academy Infectious Disease Foundation, Amphia Hospital, Breda, The Netherlands; Emory University School of Medicine, UNITED STATES

## Abstract

**Introduction:**

Knowledge of within-patient dynamics of resistance plasmids during outbreaks is important for understanding the persistence and transmission of plasmid-mediated antimicrobial resistance. During an outbreak of a *Klebsiella pneumoniae* carbapenemase-producing (KPC) *K*. *pneumoniae*, the plasmid and chromosomal dynamics of *K*. *pneumoniae* within-patients were investigated.

**Methods:**

During the outbreak, all *K*. *pneumoniae* isolates of colonized or infected patients were collected, regardless of their susceptibility pattern. A selection of isolates was short-read and long-read sequenced. A hybrid assembly of the short-and long-read sequence data was performed. Plasmid contigs were extracted from the hybrid assembly, annotated, and within patient plasmid comparisons were performed.

**Results:**

Fifteen *K*. *pneumoniae* isolates of six patients were short-read whole-genome sequenced. Whole-genome multi-locus sequence typing revealed a maximum of 4 allele differences between the sequenced isolates. Within patients 1 and 2 the resistance gene- and plasmid replicon-content did differ between the isolates sequenced. Long-read sequencing and hybrid assembly of 4 isolates revealed loss of the entire KPC-gene containing plasmid in the isolates of patient 2 and a recombination event between the plasmids in the isolates of patient 1. This resulted in two different KPC-gene containing plasmids being simultaneously present during the outbreak.

**Conclusion:**

During a hospital outbreak of a KPC-producing *K*. *pneumoniae* isolate, plasmid loss of the KPC-gene carrying plasmid and plasmid recombination was detected within the isolates from two patients. When investigating outbreaks, one should be aware that plasmid transmission can occur and the possibility of within- and between-patient plasmid variation needs to be considered.

## Introduction

Recent years have shown a rise in *Klebsiella Pneumoniae* carbapenemase (KPC)-producing *Klebsiella pneumoniae* worldwide [1]. Infections with KPC-producing *K*. *pneumoniae* are associated with increased mortality and an increased length of hospital stay [2,3]. Moreover, nosocomial infections and colonization with KPC-producing *K*. *pneumoniae* are known to be an important source for its transmission within and between health care facilities [4,5]. Prolonged carriage of KPC-producing *K*. *pneumoniae* has been described and several risk factors associated with an increased duration of colonization have been identified [6]. The gene encoding the KPC enzyme in *K*. *pneumoniae* (*bla*KPC) is generally located on large conjugative plasmids which can undergo multiple rearrangements during long-term patient colonization [7,8]. Studies investigating the dynamics of *bla*KPC- plasmids in *K*.*pneumoniae* isolates during colonization only include the KPC-producing (or carbapenem-resistant) isolates [8,9]. However, when only *bla*KPC containing *K*.*pneumoniae* isolates (KPC-KP) are included in the analysis, loss of the KPC enzyme encoding plasmid itself cannot be detected. Moreover, studies on *bla*KPC-plasmid dynamics within-patients during an outbreak remain limited, especially in countries with a low prevalence of *bla*KPC-plasmid carriage [10].

Analysing resistance plasmids encoding the KPC enzyme is typically performed using a combination of short- and long-read whole-genome sequencing of an isolate [8,9,11]. Repeat sequences prohibit the complete assembly of the bacterial chromosome and plasmids using short-read sequence data only, resulting in separate contigs of which the origin, plasmid or chromosome, is unknown. [12]. Current automated algorithms aiming to reconstruct plasmids from short-read sequence data are not able to correctly construct large resistance plasmids [12].

In 2017 an outbreak occurred of a KPC-KP in a teaching hospital in Tilburg, the Netherlands. During this period, in all patients colonized or infected with a KPC-producing *K*. *pneumoniae*, *K*. *pneumoniae* isolates were collected. To investigate the within-patient plasmid and chromosomal dynamics during this outbreak a selection of isolates was sequenced and a plasmid analysis was performed using a hybrid assembly of short- and long-read sequence data.

## Method

### *Klebsiella pneumoniae* collection

**Fig 1:**
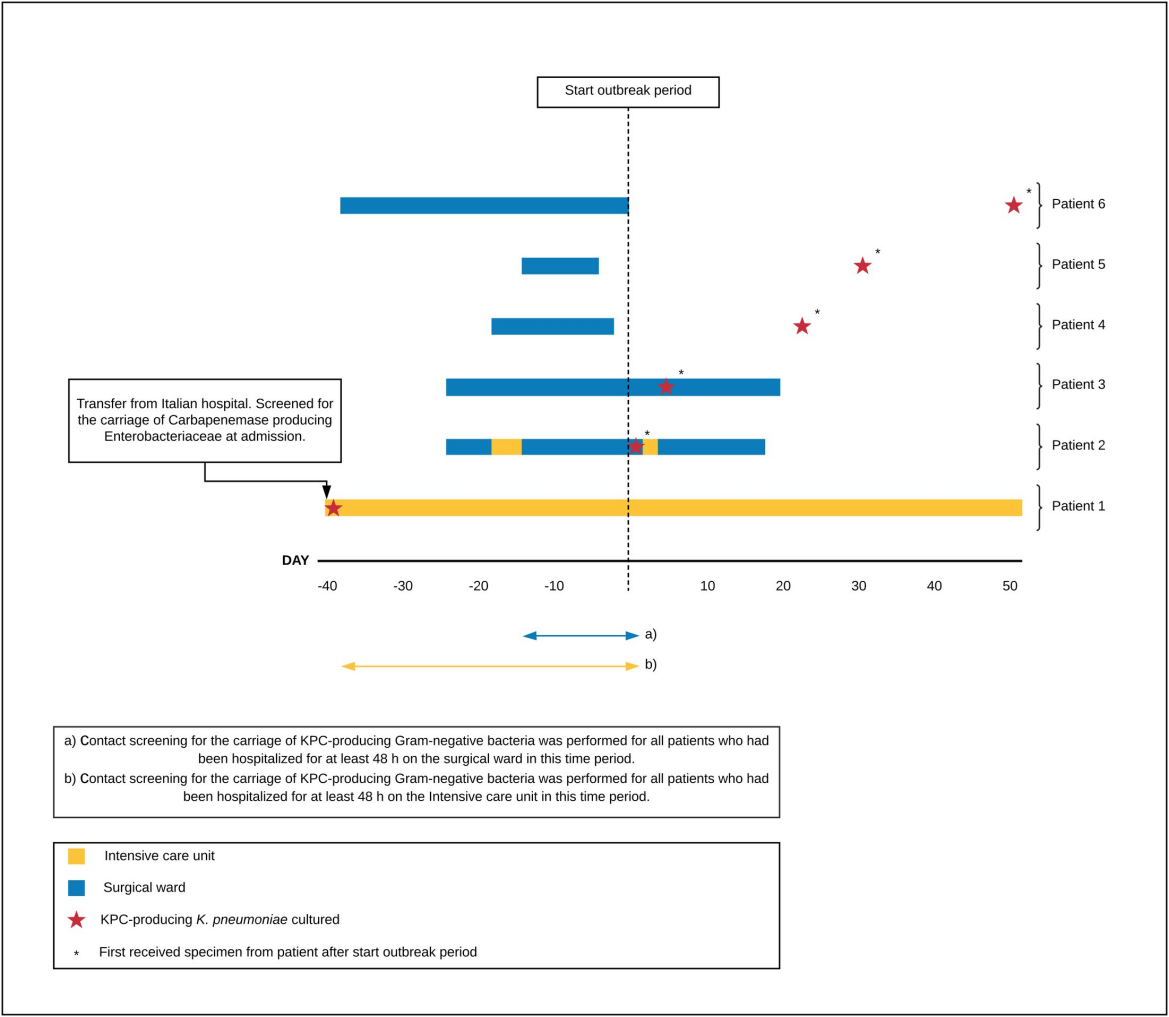
Timeline graph with the ward each patient was admitted on previous to and during the outbreak period and the day of the first cultured KPC-KP of each patient detected in the outbreak.

From 22 October 2017 until 31 December 2017 an outbreak of a KPC-KP occurred in the intensive care unit and surgical ward of a 796-bed teaching hospital in Tilburg, the Netherlands (**Fig 1**). The outbreak was recognized on the 22^nd^ of October 2017, when a KPC-KP was detected in a urine sample of a patient (patient 2) admitted on the surgical ward. This event followed the earlier repatriation of a patient (the index patient 1) from an Italian hospital on the 11^th^ of September 2017, who was found to be colonized with KPC-KP two days after admission to the intensive care unit (**Fig 1**). Because carbapenem-resistant Enterobacteriaceae have been practically absent in this hospital so far and because patient 2 was also admitted on the intensive care unit previous to the detection of a KPC-KP in the patient’s urine sample (3–7 October), the finding was considered suspect for nosocomial transmission (**Fig 1**). An outbreak management team was formed and in both the surgical ward and intensive care unit patient contacts were screened for KPC-KP carriage (**Fig 1**). During this outbreak, a total of 6 patients were colonized (n = 4) or infected (n = 2) with a KPC-KP. In all patients, for every specimen wherein a *K*. *pneumoniae* isolate was obtained, an isolate, regardless of the susceptibility pattern, was collected and stored at -80C° using Microbank^TM^. Species identification was performed using the Bruker MALDI Biotyper^TM^ (BD Diagnostics, MD, USA), and antimicrobial susceptibility testing was performed using the Phoenix^TM^ platform (BD Diagnostics, MD, USA) and EUCAST breakpoints v.9.0. [13]. Every specimen, from which a *K*. *pneumoniae* isolate was obtained that was measured susceptible to meropenem and/or imipenem, was additionally inoculated on a CHROMagar^TM^ KPC plate (CHROMagar, Paris, France).

### Short-read whole-genome sequencing

A selection of *K*. *pneumoniae* isolates was sequenced on an Illumina MiSeq using Nextera XT chemistry (Illumina, San Diego, United States) and assembled with SPAdes v. 3.9.1 [14]. The selection was made in a way that: at least each *K*. *pneumoniae* isolate with a distinct susceptibility pattern and at least one *K*. *pneumoniae* isolate per patient per specimen type was sequenced. A distinct susceptibility pattern was defined as a four-fold difference in minimal inhibitory concentration (MIC) in any of the following antibiotics: amoxicillin-clavulanic acid, ceftriaxone, ceftazidime, meropenem, ciprofloxacin, and gentamicin. Before sequencing isolates were regrown and plated on a CHROMagarTM KPC plate (CHROMagar, Paris, France) when measured resistant to meropenem and on sheep blood agar when measured susceptible to meropenem. Plates were incubated for 18 to 24 hours at 35 to 37C°. The DNA isolation and sequencing protocol are described in the **S1 Data**. The following quality control criteria for acceptable assemblies were used: coverage: ≥20; number of scaffolds: ≤1000; N50: ≥15.000 bases and maximum scaffold length: ≥50.000 bases.

### Short-read whole genome analysis

Whole-genome MLST (wgMLST) (core and accessory genome) was performed for all sequenced isolates using Ridom SeqSphere+, version 4.1.9 (Ridom, Münster, Germany). Species-specific typing schemes were used as described by Kluytmans-van den Bergh et al. [15]. The all-to-all pairwise genetic difference was calculated between the isolates by counting the total number of allele differences in the wgMLST typing scheme and by dividing the total number of allele differences in the wgMLST typing scheme by the total number of shared alleles in the wgMLST typing scheme, ignoring pairwise missing values. The phylogenetic tree was visualized using iTOL v5.5.1 [16]. The genomes of the sequenced isolates were uploaded to the online bioinformatic tools ResFinder v.3.1 and PlasmidFinder v.2.0 (Center for Genomic Epidemiology, Technical University of Denmark, Lingby, Denmark) [17,18]. Acquired resistance genes were called when at least 60% of the length of the best matching gene in the ResFinder database was covered with a sequence identity of at least 90%. Plasmid replicon genes were called when at least 60% of the sequence length of the replicon gene in the PlasmidFinder database was covered with a sequence identity of at least 80%.

### Long-read whole genome sequencing

A selection of the isolates was long-read sequenced on a MinION sequencer using the FLO-MIN106D flow cell and the Rapid Barcoding Sequencing Kit SQK RBK004 according to the standard protocol provided by the manufacturer (Oxford Nanopore Technologies, Oxford, United Kingdom). The selection was made in a way that in each patient in which more than one isolate was short-read sequenced all isolates with a unique plasmid replicon content were long-read sequenced. Short-and long-read sequencing was performed from extracted DNA of the same regrown culture.

### Hybrid short- and long-read plasmid analysis

A hybrid assembly of long-read and short-read sequence data was performed using Unicycler v.0.8.4 [19]. The genomes created using the hybrid assembly were uploaded to the online bioinformatic tools ResFinder v.3.1 and PlasmidFinder v.2.0 (Center for Genomic Epidemiology, Technical University of Denmark, Lingby, Denmark) [17,18]. Contigs created by the hybrid assembly that were smaller than 1000kb and that contained plasmid replicons were extracted from the assembly graph using BANDAGE v.0.8.1 [20]. All extracted plasmid contigs were annotated using Prokka v. 1.13.3 [21]. A pan-genome was constructed and pairwise comparisons were performed of plasmids between isolates of the same patient using BLAST+ v.2.6.0. (identity cut-off 95%) and Gview v.1.7. via the Gview webserver (https://server.gview.ca/) [22,23].

### Accession numbers

Generated raw reads were submitted to the European Nucleotide Archive (ENA) of the European Bioinformatics Institute (EBI) under the study accession number: PRJEB35018 (link to data: https://www.ebi.ac.uk/ena/data/view/PRJEB35018).

## Results

### *Klebsiella pneumoniae* collection

**Fig 2:**
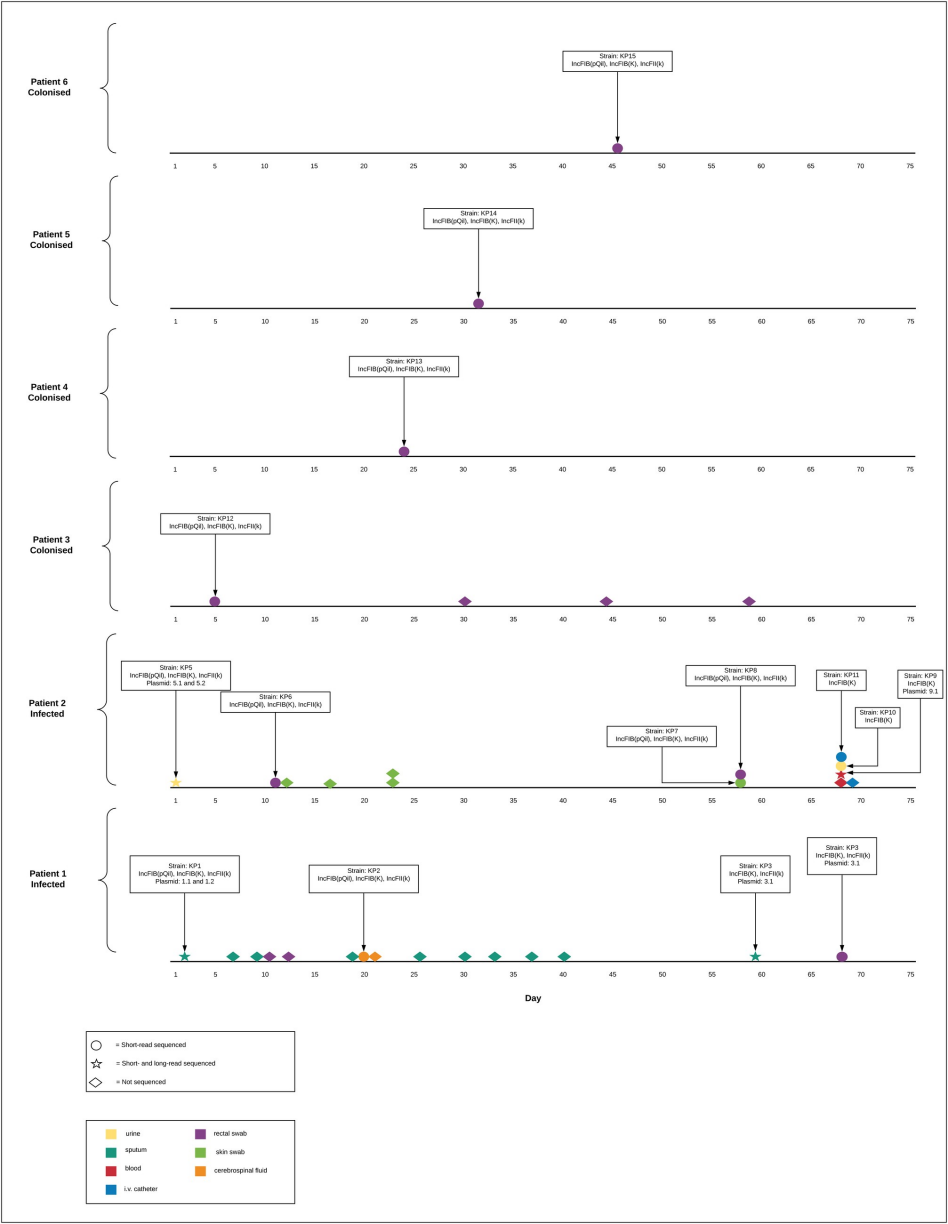
Timeline graphic containing the collected isolates per patient during the study period.

During the outbreak period, a total of 35 *K*. *pneumoniae* isolates (patient 1: n = 15, patient 2: n = 13, patient 3: n = 4 and patient 4–6: n = 1) with two distinct susceptibility patterns were collected(**Fig 2; S1 Table**). The two distinct susceptibility patterns were detected in isolates cultured from patient 2 only and were based on differences in MIC for amoxicillin-clavulanic acid, ceftriaxone, ceftazidime, and meropenem (**Table 1**). In the specimens containing a *K*. *pneumoniae* isolate susceptible to meropenem, no growth was detected on the CHROMagar^TM^ KPC plate. Fifteen isolates were selected to be sequenced: patient 1: n = 4, patient 2: n = 7, patient 3–6: n = 1 (**Fig 2; Table 1**).

**Table 1:** *K*. *pneumoniae* isolates used for short-read whole-genome sequencing.

Patient	Isolate	Susceptibility profile	Sequence type^¥^	Specimen	Date culture^+^	MIC (mg/L)*					
						amcl	cftz	cftr	mero	cipr	gent
1	KP1	1	307	Sputum	2	>32	>16	>4	>8	>1	>4
1	KP2	1	307	Cerebrospinal fluid	20	>32	>16	>4	>8	>1	>4
1	KP3	1	307	Sputum	59	>32	>16	>4	>8	>1	>4
1	KP4	1	307	Rectal swab	68	>32	>16	>4	>8	>1	>4
2	KP5	1	307	Urine	1	>32	>16	>4	>8	>1	>4
2	KP6	1	307	Rectal swab	11	>32	>16	>4	>8	>1	>4
2	KP7	1	307	Skin swab	58	>32	>16	>4	>8	>1	>4
2	KP8	1	307	Rectal swab	58	>32	>16	>4	>8	>1	>4
2	KP9	2	307	Blood	68	4	< = 0,5	< = 0,5	< = 0,25	>1	>4
2	KP10	2	307	Urine	68	4	< = 0,5	< = 0,5	< = 0,25	>1	>4
2	KP11	2	307	i.v. catheter	68	4	< = 0,5	< = 0,5	< = 0,25	>1	>4
3	KP12	1	307	Rectal swab	5	>32	>16	>4	>8	>1	>4
4	KP13	1	307	Rectal swab	24	>32	>16	>4	>8	>1	>4
5	KP14	1	307	Rectal swab	31	>32	>16	>4	>8	>1	>4
6	KP15	1	307	Rectal swab	46	>32	>16	>4	>8	>1	>4

*MIC testing was performed using the BD Phoenix^™^. Amcl: amoxicillin-clavulanic acid; cftr: ceftriaxone; cftz: ceftazidime;mero: meropenem; cipr: ciprofloxacin; gent: gentamicin.

^+^ Day culture from study start at 22–10 (day 1).

^¥^ Based on multi-locus sequence typing scheme of Institut Pasteur, France.

### Short-read whole genome analysis

Short-read WGS was performed on 15 *K*. *pneumoniae* isolates of 6 patients (**Fig 2**). Despite MIC testing revealing two distinct susceptibility patterns, using wgMLST the maximum number of allele differences detected between the various isolates was 4 (0.09%)(**S1 Fig; S2 Table**). Moreover, in none of the pairwise comparisons of the sequenced isolates did the number of allele differences exceed the limit of clonal relatedness (smaller or equal to 0.45%) as defined by Kluytmans-van den Bergh et al. (**S1 Fig**) [15]. The acquired resistance gene content did differ most notably with four isolates not containing a *bla*KPC gene in the Whole Genome Assembly (WGA) (**Table 2**), explaining the difference in antimicrobial susceptibility profile seen between the isolates. Moreover, 2 isolates contained a tet(A) gene not detected in any of the other genomes (**Table 2**). Plasmid replicon content also differed between the isolates: three isolates contained one plasmid replicon gene, three isolates contained two plasmid replicon genes and nine isolates contained three plasmid replicon genes. The difference in plasmid replicon- and acquired resistance gene-content was shown between isolates collected from the same patient (both in isolates from patient 1 and patient 2) (**Tables 1** and **2**).

**Table 2:** Acquired resistance gene- and plasmid replicon-content of whole-genome assembly of the sequenced *K*. *pneumoniae* isolates.

Patient	isolate	Plasmid replicon and acquired resistance gene content whole-genome assembly	
		Resistance genes^^^	Plasmid replicons
1	KP1	aac(6')Ib-cr, blaKPC-3, blaOXA-1, blaOXA-9-like, blaTEM-1A-like, catB3-like, QnrB66-like	IncFIB(pQil), IncFIB(K), IncFII(K)
1	KP2	aac(6')Ib-cr, blaKPC-3, blaOXA-1, blaOXA-9-like, blaTEM-1A-like, catB3-like, QnrB66-like, tet(A)	IncFIB(pQil), IncFIB(K), IncFII(K)
1	KP3	aac(6')Ib-cr, blaKPC-3, blaOXA-1, blaSHV-28, catB3-like, tet(A)	IncFIB(K), IncFII(K)
1	KP4	aac(6')Ib-cr, blaKPC-3, blaOXA-1, blaSHV-28, catB3-like, tet(A)	IncFIB(K), IncFII(K)
2	KP5	aac(6')Ib-cr, blaKPC-3, blaOXA-1, blaOXA-9-like, blaTEM-1A-like, catB3-like	IncFIB(pQil), IncFIB(K), IncFII(K)
2	KP6	aac(6')Ib-cr, blaKPC-3, blaOXA-1, blaOXA-9-like, blaTEM-1A-like, catB3-like	IncFIB(pQil), IncFIB(K), IncFII(K)
2	KP7	aac(6')Ib-cr, blaKPC-3, blaOXA-1, blaOXA-9-like, blaTEM-1A-like, catB3-like	IncFIB(pQil), IncFIB(K), IncFII(K)
2	KP8	aac(6')Ib-cr, blaKPC-3, blaOXA-1, blaOXA-9-like, blaTEM-1A-like, catB3-like	IncFIB(pQil), IncFIB(K), IncFII(K)
2	KP9		IncFIB(K)
2	KP10		IncFIB(K)
2	KP11		IncFIB(K)
3	KP12	aac(6')Ib-cr, blaKPC-3, blaOXA-1, blaOXA-9-like, blaTEM-1A-like, catB3-like	IncFIB(pQil), IncFIB(K), IncFII(K)
4	KP13	aac(6')Ib-cr, blaKPC-3, blaOXA-1, blaOXA-9-like, blaTEM-1A-like, catB3-like	IncFIB(pQil), IncFIB(K), IncFII(K)
5	KP14	aac(6')Ib-cr, blaKPC-3, blaOXA-1, blaOXA-9-like, blaTEM-1A-like, catB3-like	IncFIB(pQil), IncFIB(K), IncFII(K)
6	KP15	aac(6')Ib-cr, blaKPC-3, blaOXA-1, blaOXA-9-like, blaTEM-1A-like, catB3-like	IncFIB(pQil), IncFIB(K), IncFII(K)

^^^All isolates contained the following resistance genes: aac(3)-IIa-like, blaSHV-28, dfrA14-like, fosA-like, oqxA-like, oqxB-like.

### Hybrid short- and long-read plasmid analysis

**Fig 3:**
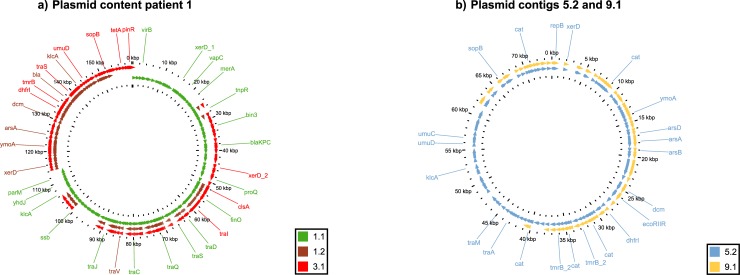
a) Gview BLAST plasmid comparison of all annotated plasmids in patient 1. b) Plasmid comparison of annotated plasmids 5.2 and 9.1 of patient 2. Each arrow represents a coding sequence and not necessarily transcriptional direction; Gene names are depicted as generated by prokka.

Four isolates were selected, based on within-patient plasmid replicon content differences, to be long-read sequenced: KP1 and KP3 of patient 1 and KP5 and KP9 of patient 2 (**Fig 2**). Hybrid assembly of short- and long-read sequences revealed 10 contigs of which, based on size, 6 were assumed to be of plasmid origin (**Table 3**). Despite the fact that only one IncFII(k) replicon was detected in the short-read whole-genome assembly of isolate KP1, the hybrid assembly revealed that the IncFII(K) plasmid replicon was actually present in two separate plasmids in the KP1 isolate: an IncFII(K) and IncFIB(K) replicon plasmid and an IncFII(K)and IncFIB(pQil) replicon plasmid. Within patient 1, GView BLAST analysis revealed the 3.1 plasmid to be a recombinant plasmid resulting from a recombination event between 18 CDS of plasmid 1.1 (containing the *bla*KPC gene), 50 CDS present in plasmid 1.1 and plasmid 1.2, 56 CDS of plasmid 1.2 only combined with an introduction of a tet(A) gene containing transposon (**Fig 3A**). Moreover, it revealed a loss of major parts of the plasmid content between isolates KP1 and KP3 without affecting the isolates susceptibility pattern. In patient 2, the loss of the entire 5.1 *bla*KPC gene-containing plasmid was observed between isolates KP5 and KP9 (**Table 3**). Additionally, a 26.786 bp deletion occurred in plasmid 5.2 when compared to plasmid 9.1 resulting in loss of 34 coding sequences among which were the antibiotic resistance genes aac(6')Ib-cr, *bla*OXA-1, and a catB3-like gene (**Fig 3B; Table 3**).

**Table 3:** Size, gene-, acquired antimicrobial resistance gene-and plasmid replicon-content of the plasmid contigs created with the hybrid assemblies.

isolate	Plasmid	Plasmid contig size (bp)	Number of CDS* in plasmid contig	Plasmid replicon and acquired resistance gene content plasmid construct	
				Resistance genes	Plasmid replicons
KP1	1.1	114416	129	blaKPC-3, blaOXA-9-like, blaTEM-1A-like	IncFIB(pQil), IncFII(K)
KP1	1.2	102547	111	aac(3)-IIa-like, aac(6')Ib-cr, blaOXA-1, catB3-like, dfrA14-like	IncFIB(K), IncFII(K)
KP3	3.1	129321	138	aac(3)-IIa-like, aac(6')Ib-cr, blaKPC-3, blaOXA-1, catB3-like, dfrA14-like, tet(A)	IncFIB(K), IncFII(K)
KP5	5.1	114416	130	blaKPC-3, blaOXA-9-like, blaTEM-1A-like	IncFIB(pQil), IncFII(K)
KP5	5.2	68609	80	aac(3)-IIa-like, aac(6')Ib-cr, blaOXA-1, catB3-like, dfrA14-like	IncFIB(K)
KP9	9.1	41823	46	aac(3)-IIa-like, dfrA14-like	IncFIB(K)

*CDS: coding sequences

## Discussion

The plasmid replicon content of the first sequenced *bla*KPC containing isolate of each patient during the hospital outbreak was similar between the different patients. However, during the outbreak within both patient 1 and patient 2 the plasmid replicon contents highly varied. This variation in plasmid replicon content was partially the result of plasmid loss observed in both patients, leading to a distinct susceptibility pattern in the isolates of one of these patients. A previous study also described plasmid loss during long time colonization in *K*. *pneumoniae* [9]. However, the present study also includes isolates with all distinct resistance patterns revealing the loss of a *bla*KPC containing plasmid within an outbreak setting. The similar replicon content of the plasmids 1.1, 1.2 and 5.1 would suggest that incompatibility between these plasmids [24,25] was the cause of the plasmid loss observed in the isolates of patient 1 and 2. Besides plasmid loss, within-patient 1 acquisition of a tet(A) containing transposon was detected in the plasmid content of the KP3 isolate when compared to the plasmid content of isolate KP1. Thus not only gene loss was revealed but also the acquisition of genetic elements in the plasmid content of the isolates collected during this outbreak. The plasticity of the plasmid content in bacterial isolates observed in this study has been described before both *in vitro* as *in vivo* [7,9,26]. However, recent reports also describe plasmids which remain highly stable [27,28]. This suggests that *in vivo* plasmid stability is likely the result of an interplay between host factors, plasmid content and the different plasmids composing the plasmid content of a bacterial isolate, possibly resulting in either a highly stable or unstable plasmid content.

The hybrid assembly revealed that the 3.1 plasmid was the result of a recombination event between the 1.1 and the 1.2 plasmid occurring in the KP3 isolate only (and possibly the KP4 isolate) and not in the KP5 isolate. These recombination events between different plasmids in the same isolates have also been described in other studies [9,26]. However, this is to the best of our knowledge the first study to describe within patient *bla*KPC gene-containing plasmid recombination and loss during an hospital outbreak. This recombination event led to two different *bla*KPC plasmids occurring within one patient and plasmid transmission of these two different plasmids could have occurred during this outbreak. Several studies have already reported *bla*KPC-plasmid transmission between different isolates during outbreaks [11,29]. Distinguishing outbreak related from non-outbreak related plasmids based on sequence data is essential for using molecular data to confirm *bla*KPC-containing plasmid transmission in outbreaks. Our findings suggest that when investigating plasmid transmission during outbreaks, the possibility of within-patient plasmid variation needs to be considered. Therefore, it could well be that transmission of *bla*KPC-containing IncF plasmids within hospital outbreaks cannot be dismissed based on sequence dissimilarity between the different plasmids investigated. Complicating the investigation of plasmid transmission during hospital outbreaks even when the antibiotic susceptibility pattern is not altered.

The present study has some limitations. The first was that in a culture of a specific specimen, colonies of the same morphology were not routinely isolated and stored. Therefore, possible subpopulations were not detected. Despite this, in the cultures in which a *K*. *pneumoniae* was isolated that was measured susceptible to carbapenem, no growth was observed on the CHROMagar KPC^TM^ suggesting that no resistant subpopulations were present in these specimens. Based on susceptibility pattern differences, specimen type of isolation, and plasmid replicon content a selection of the isolates were sequenced therefore plasmid variations that did not influence the susceptibility pattern, specimen type of isolation, and plasmid replicon content might go undetected. Moreover, since only 1 isolate was collected in patients 3–6 no conclusions can be drawn regarding longitudinal plasmid variation in these patients.

Concluding, during a hospital outbreak of a *bla*KPC producing *K*. *pneumoniae* isolate plasmid loss of the *bla*KPC carrying plasmid and plasmid recombination was detected in two patients. When investigating outbreaks wherein plasmid transmission can occur, the possibility of within- and between-patient plasmid variation needs to be considered.

## Supporting information

S1 Data Supplementary materials and methods. (DOCX)

S1 Table Susceptibility testing results of the collected *K*. *pneumoniae* isolates. (XLSX)

S2 Table Distance matrix of allele differences (% of allele differences) between the isolates sequenced in this study as determined using wgMLST. (XLSX)

S3 Table Table A response to reviewers. (XLSX)

S1 Fig Neighbour joining tree based on the wgMLST analysis of the different isolates. A publicly available genome of a K. pneumoniae sequence type (ST) 256 strain (ATCC^®^ BAA-1705TM) was included in the tree as reference for genetic distance.
(EPS)
