# Supplementary material for: Within-patient plasmid dynamics in Klebsiella pneumoniae during an outbreak of a carbapenemase-producing Klebsiella pneumoniae
Source: PLoS One. 2020 May 18;15(5):e0233313. doi: 10.1371/journal.pone.0233313 (PMC7233586; doi:10.1371/journal.pone.0233313)
Supplement: S1 Data — (DOCX) [file pone.0233313.s001.docx]

**DNA isolation, library preparation, and DNA sequencing**

Colonies were suspended in 1000 µL TE buffer. Bacterial DNA was extracted using MagNA Pure LC Total Nucleic Acid Kit - High Performance on a MagNA Pure LC instrument (Roche Diagnostics International Ltd, Rotkreuz, Switzerland) according to the manufacturer’s Protocol. A total of 200 µL bacterial solution was used for DNA extraction, together with Lysis/Binding buffer and proteinase K according to kit-protocol, with an elution volume of 100 µL. DNA concentration was normalized to 0.2 ng/µl by adding TE buffer to eluate. DNA samples were quantified using the Qubit Fluorometer dsDNA 3.0 system (Thermofisher Scientific, Waltham, MA, USA). A fragmented genomic DNA library was prepared using a NexteraXT DNA sample preparation kit. Library purification was performed using Agencourt AMPure XP beads. Subsequent sequencing was conducted in a paired-end 2 x 300 bp mode using an Illumina MiSeq sequencer (Illumina, San Diego, CA, USA).
